# Gene expression profiling in the striatum of inbred mouse strains with distinct opioid-related phenotypes

**DOI:** 10.1186/1471-2164-7-146

**Published:** 2006-06-13

**Authors:** Michal Korostynski, Dorota Kaminska-Chowaniec, Marcin Piechota, Ryszard Przewlocki

**Affiliations:** 1 Department of Molecular Neuropharmacology, Institute of Pharmacology PAS, Cracow, Poland

## Abstract

**Background:**

Mouse strains with a contrasting response to morphine provide a unique model for studying the genetically determined diversity of sensitivity to opioid reward, tolerance and dependence. Four inbred strains selected for this study exhibit the most distinct opioid-related phenotypes. C57BL/6J and DBA/2J mice show remarkable differences in morphine-induced antinociception, self-administration and locomotor activity. 129P3/J mice display low morphine tolerance and dependence in contrast to high sensitivity to precipitated withdrawal observed in SWR/J and C57BL/6J strains. In this study, we attempted to investigate the relationships between genetic background and basal gene expression profile in the striatum, a brain region involved in the mechanism of opioid action.

**Results:**

Gene expression was studied by Affymetrix Mouse Genome 430v2.0 arrays with probes for over 39.000 transcripts. Analysis of variance with the control for false discovery rate (q < 0.01) revealed inter-strain variation in the expression of ~3% of the analyzed transcripts. A combination of three methods of array pre-processing was used to compile a list of ranked transcripts covered by 1528 probe-sets significantly different between the mouse strains under comparison. Using Gene Ontology analysis, over-represented patterns of genes associated with cytoskeleton and involved in synaptic transmission were identified. Differential expression of several genes with relevant neurobiological function (e.g. GABA-A receptor alpha subunits) was validated by quantitative RT-PCR. Analysis of correlations between gene expression and behavioural data revealed connection between the level of mRNA for K homology domain containing, RNA binding, signal transduction associated 1 (*Khdrbs1*) and ATPase Na+/K+ alpha2 subunit (*Atp1a2*) with morphine self-administration and analgesic effects, respectively. Finally, the examination of transcript structure demonstrated a possible inter-strain variability of expressed mRNA forms as for example the catechol-O-methyltransferase (*Comt*) gene.

**Conclusion:**

The presented study led to the recognition of differences in the gene expression that may account for distinct phenotypes. Moreover, results indicate strong contribution of genetic background to differences in gene transcription in the mouse striatum. The genes identified in this work constitute promising candidates for further animal studies and for translational genetic studies in the field of addictive and analgesic properties of opioids.

## Background

The presence of strong genetic determinants of locomotor and analgesic response to morphine and heroin in mice was first observed more than thirty years ago [1-5]. Behavioural phenotyping of a large panel of commonly used inbred strains of mice showed tremendous diversity in the response to both acute and prolonged opioid treatments [6-9]. Strain surveys demonstrated that sensitivity to morphine is to a great degree dependent on genetic determinants. Based on a number of previous studies, we have chosen for gene expression studies four inbred mouse strains (129P3/J, SWR/J DBA/2J and C57BL/6J) with the clearest differences in opioid-related phenotypes. The 129P3/J strain failed to develop tolerance to morphine-induced analgesia [8] or physical dependence, as evidenced by the lack of withdrawal symptoms [9]. Unusual sensitivity to precipitated withdrawal [9] with extremely low morphine oral self-administration was observed in SWR/J mice [6,9]. In marked contrast, the C57BL/6J strain was found to have the highest level of oral morphine consumption [6]. However, sensitivity to the reinforcing effects of morphine in conditioned place preference and intravenous self-administration paradigms was higher in DBA mice than in C57BL [10]. The two frequently used laboratory strains of mice C57BL/6J and DBA/2J show remarkable differences in analgesic response to morphine. Moreover, several studies have reported profound differences in morphine induced locomotor activity between the sensitive C57BL/6 and insensitive DBA/2 mice [3,7].

Opioids are known to act through binding to μ-opioid receptor, which is located on GABAergic interneurons in the ventral tegmental area (VTA) and substantia nigra (SN). Mechanisms which underlie opioid actions depend on activation of dopaminergic midbrain neurons, resulting in an increased dopamine release in the mesocorticolimbic structures such as ventral and dorsal striatum [11]. The striatum, a brain region that contains high level of opioid receptors, is a major neural substrate for the locomotor and reinforcing effects of opioids [12,13]. Consequently, it is accepted that the nucleus accumbens, a region of the ventral striatum, which receives projections from the VTA, is involved in the processes of reward stimulus-response learning [14,15]. The dorsal part of the striatum, caudate-putamen, is an integral component of reward circuitry responsible for the control of motivated and motor behaviour [16,17]. The behavioural effects of opioids are associated with altered gene expression in the striatum. A single injection of morphine alters striatal expression of some genes in mice, including immediate early genes as well as transcription of cytoskeleton-related genes and proteins involved in oxidative chain [18,19]. Therefore, mechanisms that manage striatal gene expression may be implicated as important mediators in the prolonged behavioural effects of opioid administration. Elucidation of molecular mechanisms essential for action of opioids is a crucial step forward in our understanding of such phenomena as opiate reward and addiction.

Here, we have focused on the identification of the genomic factors responsible for the extreme behavioural differences outlined above. Gene expression profiles in various inbred mouse strains were previously established in multiple brain regions [20-24]. Specific genes involved in mediating the differences in running wheel activity were identified by comparing gene expression profiles in the striatum of four inbred strains (C3H/HeSnJ, 129/SvEv, C57BL/6J and 129/B6F2) [25]. Furthermore, striatal gene expression of quasi-congenic strains and their progenitors C57BL/6By and BALB/cJ were previously described and basal expression of aldehyde dehydrogenase genes (*Aldh1a1 *and *Aldh7a1*) was suggested as the factor implicated in alcohol preference [26]. Correlation of the gene expression data with neurophenotypes across inbred strains was proposed as a method for identifying candidate genes for behavioural traits such as the level of anxiety or aggressiveness [21,24]. The combination of global gene expression and mapping analysis using recombinant inbred strains provided a method for identifying quantitative trait genes underlying complex traits [27,28]. Multidisciplinary results integrated in the WebQTL database give an opportunity to identify expression of quantitative trait loci that underlie phenotype and regulate expression of the genes selected by microarrays [29]. For example, using genetic correlation analysis and phenotype database, a positive correlation between D2 dopamine receptor (*Drd2*) expression and preference for ethanol and saccharin, as well as its negative correlation with ethanol-induced locomotor activity were found [28].

Our goal in this study was to recognize functional classes of genes and individual candidates involved in the mechanisms underlying susceptibility to the opioid actions. The gene expression profiling in combination with bioinformatic approach paves the way for investigation of genes that contribute to neurobehavioral phenotypes. Apart from diversity in the level of mRNA for a great number of genes, also differences in the appearance of putative transcript forms between the inbred mouse strains were found. The present results form the basis for further molecular and pharmacological research.

## Results

### 1. Microarray data analysis

**Figure 1:**
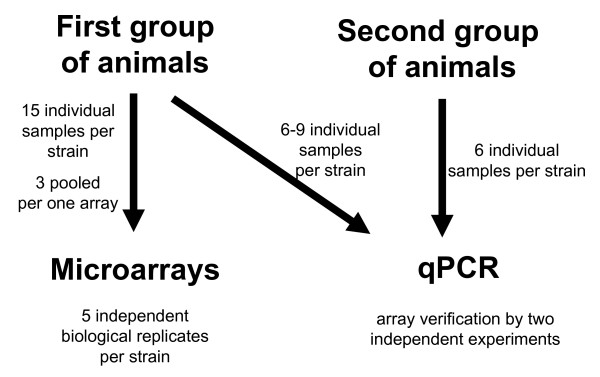
**Experimental design**. Biological replicates, sample pooling and validation of presented gene expression comparison of the four inbred mouse strains were performed using strategy described by the scheme. Five independent biological replicates of microarray were prepared for each strain of mice. Total RNA isolated from the striatum of three animals was pooled. Each pool of total RNA was separately converted to cRNA and hybridized to a single microarray. Samples from two separate groups of animals from each strain of mice were used in qPCR experiment.

Five independent biological replicates of microarray were prepared for each strain of mice. To minimize the influence of potential individual differences between the animals and technical variation introduced by tissue preparation and dissection, total RNA isolated from the striatum of three animals was pooled. Each pool of total RNA was separately converted to cRNA and hybridized to a single microarray (Figure 1). Microarray replicates were very similar: the Pearson's product-moment correlation coefficient among the arrays ranged between: 0.98–0.99 for 129P3/J, 0.97–0.99 for C57BL/6J, 0.97–0.99 for DBA/2J and 0.98–0.99 for SWR/J (using results from PDNN normalization method, see below). The data was pre-processed to correct for systematic differences between the arrays. There is still no standard protocol for pre-processing of Affymetrix array data. However, the process of normalization has a profound influence on detection of differentially expressed genes. Results from three widely acceptedalternative methods:PDNN (Position-Dependent Nearest Neighbor), RMA (Robust Multichip Analysis) and MBEI (Model-Based Expression Index) were used for further analysis. The above-mentioned methods use different algorithms and assumptions so they produce different estimates of transcripts abundance. RMA provides statistically robust averaging methods, MBEI is the weighted average of PM/MM differences and PDNN fits a model that allows sequence-specific binding affinities and nearest-neighbor stacking interactions. Pairwise Pearson's correlation coefficient of expression level between methods ranged from 0.89 (PDNN and RMA) to 0.82 (PDNN and MBEI). The performed analysis takes into account the level of agreement between the methods and promotes transcripts with significant differences in all of them. The final list of differentially expressed transcripts includes 1000 transcripts selected by all three methods, 454 transcripts selected by at least two methods and only 74 transcripts selected by one method.

### 2. Differences in gene expression in the striatum among the four inbred mouse strains

**Figure 2:**
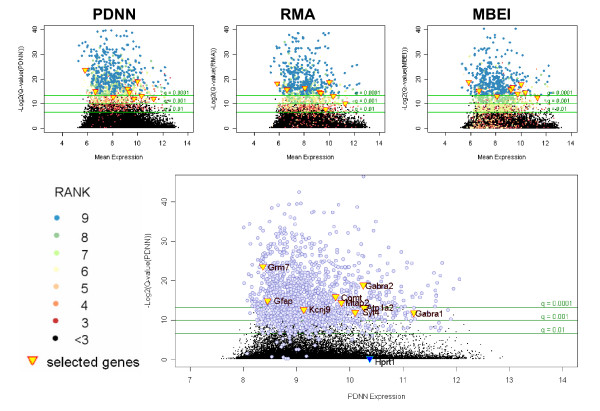
**Distribution of ranks according to the three methods of array pre-processing (PDNN, RMA and MBEI)**. Significance of inter-strain differences is presented as a plot of logarithm of q-value versus expression level. Particular ranks are marked with colour as indicated on the left. Significance steps for ranks are marked with green horizontal lines. 1528 significant probe-sets are presented below as dots marked with light blue. Additionally, selected for qPCR genes were marked with yellow triangles with gene symbols (*Atp1a2 *– 1455136_at, *Comt *– 1418701_at, *Gabra1 *– 1436889_a, *Gabra2 *– 1455444_a, *Grm7 *– 1459532_at, *Kcnj9 *– 1450712_at, *Syt4 *– 1415844_at, *Gfap *– 1440142_s_at, *Mtap2 *– 1440699_at, *Hprt1 *– 1448736_a_at).

Gene expression values were filtered to remove probe-sets with hybridization signal close to the background noise level. The remaining resulting in 24.508 probe-sets (54.3% of all probe-sets on the array) were taken into further investigation. One-way ANOVA with the mouse strain as the factor has generated p-value for each of the 24.508 probe-sets. Taking into account the fact that during ANOVA, a dozen thousands of hypotheses are being tested simultaneously, the q-value – method which provides a measure of statistical significance for each individual probe-set was used. Whereas the p-value is a measure of significance in terms of the false positive rate, the q-value is a measure in terms of the false discovery rate (FDR). Here, FDR is the expected proportion of false positive results among all rejected hypotheses multiplied by the probability of making at least one rejection [30]. The threshold for a significant difference was set at three q-value cut-offs (q < 0.01, q < 0.001, q < 0.0001) for results obtained from the three different methods of array normalization process (Figure 2). The list of differentially expressed genes was established using similar approach as the previously described *level of agreement *[31]. The obtained results were used to create a rank for each probe-set (see Methods). Differences between 21.972 (90%) probe-sets did not reach the level of significance, while 344 (1.4%) probe-sets reached the highest rank. 1528 (6.2%) probe-sets reached at least rank 3 and were selected for further bioinformatic analyses (Figure 2). The probe-sets targeted 1460 different transcripts (~3% of transcripts targeted by the array), including about 520 expressed sequence tags (ESTs). The complete list of probe-sets is included in additional material (see Additional file 2).

### 3. Gene ontology analysis

Differentially expressed genes were investigated for putative cellular functions. Significantly over-represented functional groups of genes were obtained from gene ontology (GO) DAVID 2.1 database . Analysis of biological function of genes whose expression differed between the strains revealed over-representation of those related to protein biosynthesis (42 genes) and carboxylic acid metabolism (28 genes). Candidates genes with potential involvement in the neuronal function were found among genes classified to over-represented groups linked to vesicle-mediated transport (25 genes), microtubular cytoskeleton (31 genes) and synaptic transmission (15 genes). Furthermore, cellular localization of two other over-represented groups of transcripts was linked to mitochondrion (64 genes) and to ribosome (22 genes) (Table 1).

Functional classification of strain-specific gene expression profiles was also performed. Significant over-representation in C57BL/6J mice was found for three ontology groups of genes were significantly over-represented, including: cytoskeleton organization (11 genes) and negative regulation of cell organization (3 genes). Specific functional groups associated with lipid metabolism (11 genes) and carboxylic acid metabolism (9 genes) were found to be over-represented in DBA/2J. On the other hand, genes connected with regulation of apoptosis (9 genes) were over-represented in SWR/J strain. Using uniform rigorous criteria for GO analysis, no over-represented functional classes for 129P3/J were found. Detailed description of over-represented groups with gene lists are in Table 2 and additional material (see Additional file 3).

### 4. qPCR and analysis of multiple array probe-sets

Quantitative RT-PCR (qPCR) was performed on samples from individual animals, and was used to verify the differential expression detected by microarrays. Five genes (*Atp1a2*, *Comt*, *Gabra1*, *Gabra2 *and *Syt4*) were selected for a qPCR experiment since they were linked by GO analysis to synaptic transmission (GO:0007268). Group III metabotropic glutamate receptor (*Grm7*) and a subunit of G protein activated inward rectifier potassium channel (*Kcnj9*) are also involved in neuronal transmission [32]. In addition, two cytoskeletal genes *Gfap *and *Mtap2 *were selected due to evidences of their regulation upon chronic morphine treatment [33,34]. Each qPCR assays target mRNA sequence in the coding region (Table 3). To detect existence of alternative mRNA forms, signal intensity from multiple probe-sets designed to detect the same transcript was compared with qPCR results. To confirm biological differences between the strains, additional qPCR experiment was performed on independent RNA samples.

**Figure 3:**
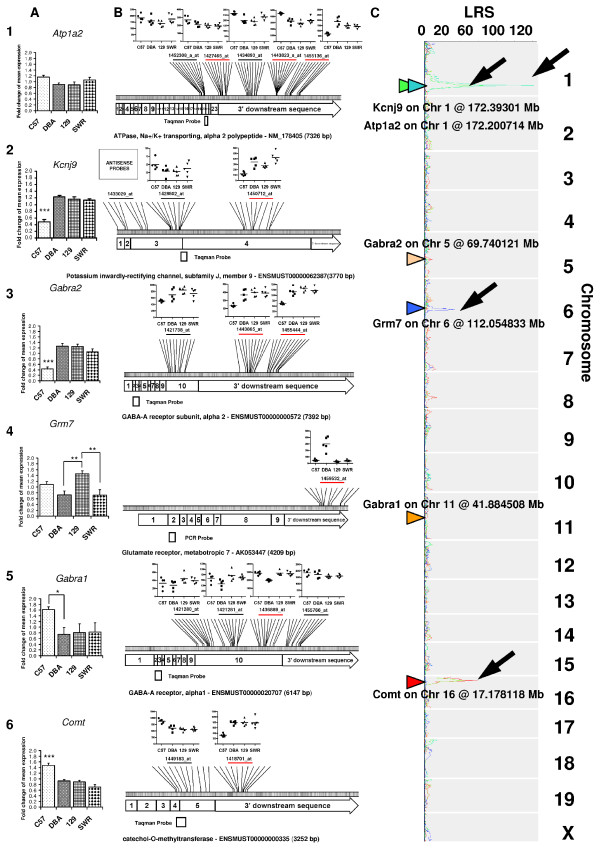
**Analyses of gene expression of the six candidate genes by qPCR method (A), multiple probe-sets from the microarray (B) and genome-wide interval mapping (C)**. **A**. Results of qPCR are presented as fold change of average expression level across all the four mouse strains. Transcript region covered by Taqman probe is marked in the scheme of transcript structure. Error bars represents ± standard error of the mean from 6–9 individual animals. Statistical significance was determined by using ANOVA analysis followed by Tukey post hoc test (*p < 0.05, **p < 0.01, ***p < 0.001). **B**. Relative inter-strain differences in expression are presented as results from the multiple probe-sets measured by the MBEI method. Each probe was aligned with mRNA sequence and exon structure downloaded from Ensembl or GenBank databases. Image displays signal intensity from an individual array with emphasized mean value. Array probe-sets marked in red were found to be significantly different between the four strains of mice. **C**. Genome-wide interval multiple mapping for six probe-sets (*Atp1a2 *– 1455136_at, *Kcnj9 *– 1450712_at, *Gabra2 *– 1443865_at, *Grm7 *– 1459532_at, *Gabra1 *– 1436889_at, *Comt *– 1418701_at) in WebQTL (HBP/Rosen Striatum M430V2 (Apr05) PDNN Clean). The likelihood ratio statistics (LRS) score for association of the gene expression with genotype across genome of BXD recombinant strains for each probe-set is represented as coloured trace. Mouse chromosomes are plotted along y-axis, with the exception for chromosome Y. Coloured triangles indicate transcript locations. *Cis*-regulatory QTLs for *Atp1a2 *(1455136_at), *Kcnj9 *(1450712_at), *Grm7 *(1459532_at), *Comt *(1418701_at) are marked with black arrows.

**Figure 4:**
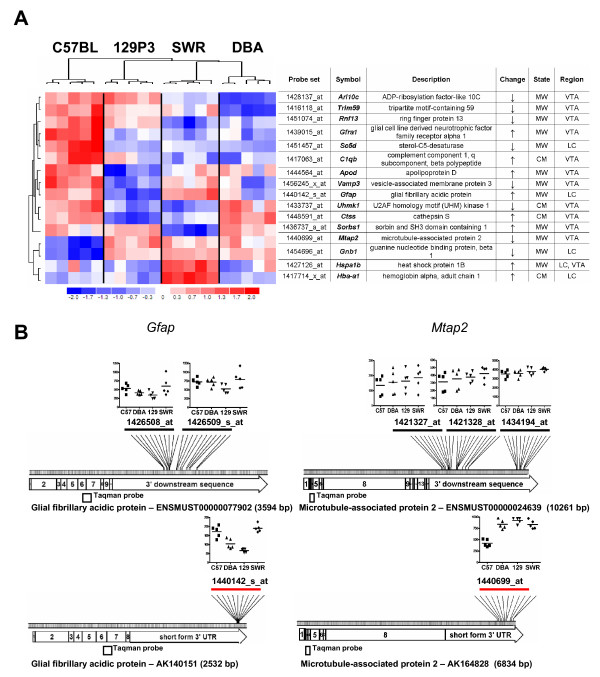
**Comparison of basal gene expression between the selected inbred mouse strains, regulated by administration in the locus ceruleus (LC) or ventral tegmental area (VTA) of the C57BL/6J mice [34]**. **A**. Cluster of genes whose expression was different according to array analysis. Coloured rectangles represent expression levels of the gene indicated on the right. Intensity of the colour is proportional to the fold change as indicated on the bar below the cluster image. The selected genes were previously found to be regulated by opioid administration as described on the right, after chronic morphine administration (CM) and morphine withdrawal (MW), respectively. Directions of the changes are indicated by arrows: (↑) – up-regulation and (↓) – down-regulation. Relative expression levels were measured by MBEI method. **B**. Expression level of *Gfap *and *Mtap2 *in the striatum, obtained by microarray experiment. Putative short mRNA forms detected by microarray probe-sets (*Gfap *– 1440142_s_at, *Mtap2 *– 1440699_at) are presented below. The image displays signal intensity from individual array with emphasized mean value.

The qPCR method found a tendency to elevation of expression level of the ATPase Na+/K+ transporting alpha 2 subunit (*Atp1a2*) transcript in C57BL/6J strain, what is in agreement with the hybridization signal measured by the probe-set 1452308_a_at covering parallel part of the mRNA sequence as the Taqman probe used in qPCR. However, the probe-set 1455136_at complementary to deep 3' untranslated region of *Atp1a2 *mRNA showed reverse profile with the lowest expression in C57BL/6J mice. The results provide indication of alternative termination of the transcription in the C57BL/6J strain, associated with higher abundance of a putative short transcript form (Figure 3B). Abundance of the potassium inwardly-rectifying channel subfamily J member 9 (*Kcnj9*) mRNA measured by microarrays was lower in C57BL/6J mice. Results of two probe-sets designed to detect 5' exons of *Kcnj9 *gene were filtered out during microarray analysis due to weak hybridization signal. qPCR validation experiment for *Kcnj9 *confirmed microarray results with a 2-fold (p < 0.001) difference in the mRNA abundance (Figure 3A). Differences in the gene expression of two GABA-A receptor subunits were confirmed using qPCR analysis. GABA-A receptor alpha1 subunit (*Gabra1*) showed ~1.5-fold higher abundance in the C57BL/6J strain (p < 0.05) compared to DBA2/J strain. On the other hand, expression of GABA-A receptor alpha2 subunit (*Gabra2*) gene in C57BL/6J strain showed opposite profile, with above 2-fold (p < 0.001) lower level compared to the other strains (Figure 3A). Signal from the microarray probe-set 1459532_at covering 3' region of metabotropic glutamate receptor 7 (*Grm7*) was detected only in DBA/2J mice (Figure 3B). To verify transcript abundance we used fluorogenic PCR probe designed to anneal to exon 2. *Grm7 *expression measured by qPCR showed significantly higher level in 129P3/J mice versus DBA/2J and SWR/J strains (p < 0.01) (Figure 3A). However, results obtained by independent qPCR experiment were not significant (Table 3). Opposite profiles of hybridization signal from two probe-sets of the catechol-O-methyltransferase gene (*Comt*) 1449183_at (covering the last 2 exons) and 1418701_at (covering deep 3' untranslated region) were found in C57BL/6J strain. qPCR method established *Comt *transcript abundance to be the highest in C57BL/6J mice, which is in agreement with the results for the probe-set 1449183_at covering the corresponding mRNA region (p < 0.001). In summary, *Comt *expression level is about 2-fold higher in C57BL/6J than in the other mouse strains, though hypothetical longer transcript form is more abundant in DBA/2J, 129P3/J and SWR/J strains (Figure 3B). Gene expression level of synaptotagmin IV (*Syt4*) was above 2-fold higher in C57BL/6J than in SWR/J strain (p < 0.01) (Table 3). qPCR analysis did not find differences in the level of glial fibrillary acidic protein (*Gfap*) and microtubule-associated protein 2 (*Mtap2*) transcripts. The differences in abundance of *Gfap *and *Mtap2 *observed between the strains are likely to be restricted to specific mRNA forms, represented on the array by the 1440142_s_at and 1440699_at probe-sets (Figure 4). Comparison between the two qPCR experiments showed significant (p < 0.0001) correlation coefficient with r = 0.92.

In order to inspect the intra-gene diversity, an interval mapping tool from the WebQTL  database was used. The negative correlation within probe-sets was observed for *Atp1a2 *(r = -0.95 between 1434893_at and 1455136_at) and *Comt *(r = -0.80 between 1449183_at and 1418701_at). Identification of possible cis- and trans-acting variations controlling gene expression was performed using HBP/Rosen Striatum M430v2 (April05) PDNN Clean dataset. The likelihood ratio statistic (LRS) provides a measure of the linkage between variation in the phenotype and genetic differences at a particular genetic locus. Significant LRS close to transcript position, which is an indication of cis-acting variations (LRS > 32.2, LOD > 7), was found for *Atp1a2 *(1434893_at, LRS = 65.48 and 1455136_at, LRS = 121.4), *Kcnj9 *(1450712_at, LRS = 65.22), *Comt *(1418701_at, LRS = 59.20 and 1449183_at, LRS = 44.95) and *Grm7 *(1459532_at, LRS = 33.51). Trans-acting elements (LRS > 9.2, LOD > 2) that are involved in expression of six analysed genes were not identified (Figure 3C).

### 5. Correlation of gene expression and phenotype

**Figure 5:**
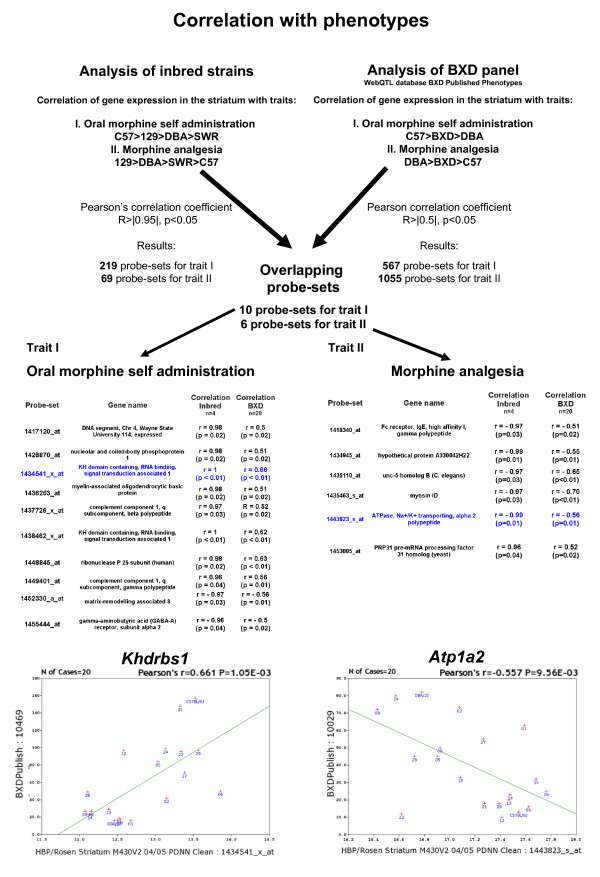
**Correlation between opioid-related phenotypes and striatal gene expression**. Comparison was performed using gene expression values of 1528 probe-sets significantly different between 129P3/J, DBA/2J, C57BL/6J and SWR/J strains [6]. Correlations between gene expression and morphine-related traits for RI BXD strains were computed using HBP/Rosen Striatum M430 v2.0 (Apr05) PDNN Clean dataset [69, 70]. All analyses were evaluated using the Pearson's correlation. Probe-sets overlapped in both analyses are presented in two tables on the left and right. Presented below scatterplots of correlation between expression values of two genes (*Khdrbs1 *and *Atp1a2*) emphasized in the table by blue colour and phenotypic data were generated by using WebQTL.

To identify genes that are accountable for the variation in response to morphine between the inbred mouse strains, differences in expression of genes selected on the basis of the microarray experiment were compared with phenotypic data. Expression values from 1528 microarray probe-sets were correlated with the results of 1) voluntary morphine consumption in two-bottle choice paradigm (C57BL/6J>129P3/J>DBA/2J>SWR/J) and 2) percent of maximal possible analgesic effects (% MPE) of 3.6 mg/kg morphine measured by the hot-plate assay (129P3/J>DBA/2J>SWR/J>C57BL/6J). Analysis resulted respectively, in 219 and 69 probe-sets with Pearson's correlation coefficient greater than |0.95| and p < 0.05. Furthermore, WebQTL database was used to correlate differentially expressed genes between BXD RI panel with the published phenotypes [29]. As in the first analysis, voluntary morphine consumption and % MPE (behavioural traits ID: 10469 and 10029) and expression results for the striatum were taken into account. Significant correlation (p < 0.05) and r > |0.5| with two morphine-related phenotypes was found for 567 and 1055 probe-sets, respectively. Results from both approaches overlapped for 10 (oral morphine self-administration) and 6 (morphine analgesia) probe-sets (Figure 5). The highest correlation with oral morphine self-administration was found for K homology domain containing, RNA binding, signal transduction associated 1 (*Khdrbs1*, 1434541_x_at). Moreover, gene expression of *Gabra2 *(1455444_at) was also found to be correlated with oral morphine consumption. Expression of *Atp1a2 *in the striatum was highly related to the analgesic potency of morphine (Figure 5). Moreover, the observed differences in gene expression of *Atp1a2 *measured by different probe-sets (1427465_at and 1443823_a_at versus 1455136_at) had reverse relation to the phenotype.

## Discussion

In this study, we have focused on the comparison of transcription profiles between four inbred strains of mice markedly differing in their sensitivity to opioids. Differences in gene expression were compared in drug-naïve animals to identify putative factors which may influence susceptibility to opioid treatment. Recent studies have described mouse strain specific gene expression profiles in the whole brain as well as in the specific regions, like the hippocampus, cerebellum, cortex or amygdala [20,21,28,35,36]. Differential abundance of transcripts was proposed to be an important factor implicated in complex behavioural traits [25]. The obtained results indicate broad differences in the transcription profile between the inbred mouse strains under investigation. Differences in the abundance of numerous transcripts, for example *Kif5b*, *Cap1*, *Pam*, *Gas5*, *Pttg1 *[20], are in agreement with the results presented in this report. Significant fraction of differences in inter-strain gene expression is independent of the brain region [35]. The presented disparity in the global transcription profiles is similar to the value estimated before (1–2% of all genes) [35]. In our case, the experiment identified more than thousand transcripts differing in abundance between the strains. A large fraction of transcripts, including nearly a half of expressed sequence tags, has not been considered so far to be connected with any specific brain function (including enzymes of basal metabolism or components of immunological system). Thus, focusing only on a few dozens of the most divergently expressed genes may lead to the situation when important factors involved in neurobehavioural variability are ignored. Gene ontology (GO) analysis was performed to identify biological themes among differentially expressed genes. Functional GO analysis showed over-representation of genes encoding proteins involved in cytoskeleton organisation and vesicle-mediated transport. That may provide partial explanation for such between-strain differences as brain weight or volume of the striatum [37]. Furthermore, expression of mRNA for several cytoskeleton-related proteins in the medial striatum of B6CBAF1/J mice was altered by acute morphine administration [18]. The observed differences in basal level of mRNA for cytoskeleton-related proteins may affect structural organization of neurons. That might be responsible for behavioural reactions to the first injection of morphine, which considerably differ between the strains under analysis [1,7,9]. This is further substantiated by the observation that in rats, chronic morphine administration changes the expression of several cytoskeleton-related genes in the striatum [33]. A group of genes connected with synaptic transmission was also identified by GO analysis. A multitude of morphine effects indicates that multiple neurotransmitter systems are involved in formation of each behavioural profile. Genes encoding two subunits of GABA-A receptor (*Gabra1 *and *Gabra2*) showed significant differences in the expression between the strains. Association of the expression of various GABA-A receptor subunits with morphine self-administration [38] and ethanol withdrawal [39] was shown previously. A switch in subunit composition of GABA-A receptor in the rat hippocampus was observed in withdrawal state from chronic intermittent ethanol treatment, with a decreased expression of alpha 1 subunit protein [40]. Gene expression of *Gabra1 *and *Gabra2 *subunits showed opposite profiles in C57BL/6J and DBA/2J mice. The idea that different subunits of GABA-A receptor are involved in mediation of different actions of the receptor provides a partial explanation of differences in morphine or ethanol preference between C57BL and DBA. In addition, a previously performed Northern Blot study on the whole brain tissue found no difference between those two strains [41], which suggests specific distinction in distribution of those transcripts in the striatum. Several lines of evidence suggest that expression of GABA-A receptor alpha subunits genes has a modulatory influence on the effects of morphine, as well as on the action of other drugs of abuse mediated by the endogenous opioid system. Catechol-O-methyltransferase (*Comt*), an enzyme degrading catecholamines, has been investigated as a candidate gene in many neurological disorders. Variation of *Comt *gene transcript level in the hippocampus between eight inbred mouse strains was found to be strongly correlated with the aggression phenotype [21]. Our results for C57BL/6J and DBA/2J are in agreement with previous observations, though the other probe-set located in the deep 3' end of transcript showed an opposite profile with high reverse correlation. One can speculate that there exist two forms of *Comt *mRNA different in length, corresponding to the long and short forms of transcript which were found in humans [42]. In humans, variability in the *Comt *gene alters the analgesic potency of morphine, demonstrating that genetic variability in non-opioid systems may indirectly influence the clinical efficacy of morphine [43]. The next interesting gene found in our research is the metabotropic glutamate receptor 7, which has been suggested to be associated with mediating aversive states and learning [44]. Deletion of *Grm7 *gene in mice produces a selective working memory impairment [45]. However, the differences observed in metabotropic glutamate receptor 7 gene expression is more difficult to interpret due to the lack of correlation between array and qPCR results. A difference in mRNA level was also observed for synaptotagmin IV (*Syt4*), a protein critical for such brain functions as learning, memory and motor coordination [46]. Two additional candidate genes: potassium inwardly-rectifying channel subfamily J member 9 (*Kcnj9*) and ATPase Na+/K+ transporting alpha 2 (*Atp1a2*) are located in the same region of chromosome 1, which is the region of formerly proposed quantitative trait loci (QTL) for basal locomotor activity [36], ethanol sensitivity [47] and ethanol withdrawal [48]. The G-protein-gated inwardly rectifying K+ channel is activated by many different substances, including opioid receptor ligands. The neuronal potassium channel subunit *Kcnj9 *knockout mice exhibited blunted morphine-induced analgesia [32] and dramatically reduced intravenous self-administration of cocaine [49]. The present results are in agreement with previous microarray data [20,36], and the differences in gene expression were confirmed by qPCR method. Cis-acting variation that caused lower expression in the C57BL strain results from polymorphism in the 5'-UTR that disrupted the binding of transcription factors [36]. Acute and chronic morphine treatment was demonstrated to modulate Na+/K+ ATPase activity in the mouse hippocampus [50]. Additionally, heterozygous *Atp1a2 *deficient mice showed augmented fear and anxiety behaviours and enhanced neuronal activity in the amygdala and piriform cortex after conditioned fear stimuli [51]. Previous studies suggested that differences in the opiate reward learning between C57BL/6J and 129/SvJ were connected to different anxiety level [52].

Interestingly, high reverse correlation among probe-sets of the same transcript was found for *Atp1a2 *and *Comt*. Inspection of hybridization profile within selected probe-sets revealed that there were no noticeable abnormalities suggesting a role of SNPs in the measurement of gene expression. Furthermore, previously estimated SNP density has shown that only a small fraction of probes contain SNPs that can affect hybridization efficiency [27,28,53]. Using mouse BLAT tool we did not identify other transcripts with high sequence homology and specificity to the probe-sets designed to detect mentioned genes. Therefore, there is no evident indication of cross-hybridization. In addition, the observed high negative correlation may suggest detection of diverse mRNA forms. In addition, for C57BL/6J and DBA/2J parental strains of BXD RI mouse panel, our results are similar to those from WebQTL database. To establish the source of these rather unexpected results, we have applied the interval mapping tool from the WebQTL database, which allowed for identification of putative cis- and trans-acting elements controlling gene expression. Differences in the signal of four genes (*Atp1a2*, *Kcnj9*, *Grm7*, *Comt*) selected for further investigation were found to be regulated by strong cis-acting elements. Cis-acting regulation of expression results from DNA variations of a gene that directly influence mRNA level of that gene [53]. This includes SNPs in the gene promoter region which could affect efficiency of transcription. Similar profile of results can be observed when inter-strain differences in abundance of alternative mRNA forms were detected by arrays. As mentioned above, results of the microarray experiment for the *Atp1a2 *and *Comt *transcripts were not consistent. We observed that, qPCR measurements were in agreement with microarray results if signal was detected in the same region of the transcript. However, general estimation of relationship between microarray and qPCR techniques was difficult due to appearance of putative mRNA forms detected by Affymetrix arrays. Most of the probes on the Affymetrix microarray are complementary to parts of the last exons or the 3' untranslated region. Differences in 3' UTRs might speak for the utilization of different signal sequences for transcription termination or alternative polyadenylation. In mice, alternative splicing was detected in 47% of genes, alternative start site and alternative transcription termination was detected in 18% and 14% genes, respectively [54]. A novel form of growth-arrest-specific transcript 5 (*Gas5*) that generate a shorter mRNA was found to be more abundant in C57BL/6J compared to 129/SvEv strain [25]. A difference in the expression of alternatively spliced forms of transcripts of κ opioid receptor gene between C57BL/6ByJ and BALB/cJ has been previously reported [55]. Moreover, a strain-dependent variation in the expression of alternatively spliced κ opioid receptor was proposed as a significant source of phenotypic variation of alcohol preference [55].

Using a combination of two independent analyses, we identified genes whose mRNA levels are highly correlated with preference to oral morphine self-administration and morphine-induced analgesia. The analysis revealed strong association between the level of mRNA for K homology domain containing, RNA binding, signal transduction associated 1 (*Khdrbs1*) and preference to morphine. The product of *Khdrbs1 *mRNA translation RNA-binding protein was implicated in gene transcription, RNA splicing and export, as well it was found to be involved in the activity-dependent regulation of dendritic mRNAs [56]. Expression level of *Gabra2 *negatively correlated with oral morphine consumption, providing another indirect evidence for involvement of GABA-A receptor in the polygenic system influencing morphine preference. Differences in *Atp1a2 *showed high correlation with phenotypic manifestation of morphine-induced analgesia measured by the hot-plate assay. Additionally, the presented data indicate alternative termination of *Atp1a2 *transcript, with putative functional role of different transcript forms.

Comparison of genes that differentiate the strains of mice with genes regulated by opioid administration in the locus coeruleus (LC) or VTA of C57BL [34] showed potential associations between basal gene transcription and expression profiles after morphine treatment and naloxone-precipitated withdrawal. Inter-strain differences in naïve mice were compared with 94 genes regulated after chronic morphine treatment or after naloxone-precipitated morphine withdrawal [34]. Genes represented by 16 probe-sets were overlapping (Figure 4). Naloxone-precipitated withdrawal induced expression of the heat shock protein 1B (*Hspa1b*). High basal heat shock protein 1B (*Hsp1b*) mRNA level in SWR strain may be connected with its unusual sensitivity to naloxone-precipitated withdrawal [9]. Induction of the heat shock protein 70 (*Hsp1b*) expression was also observed after chronic morphine treatment in mice and rats [34,38,57], and had been suggested to be associated with protection of the nervous tissue against hazardous effects of opiates [58]. Two genes connected with cytoskeleton organisation, the neuronal microtubule-associated protein 2 (*Mtap2*) and astrocytic glial fibrillary acidic protein (*Gfap*) were identified. The marker of astrocytes and astroglial activation, *Gfap*, was previously connected with morphine action [33]. However, qPCR and detailed analysis of mRNA sequence of *Gfap *and *Mtap2 *indicated that strain differences are limited to putative short transcript forms whose functional significance is not known (Figure 4).

Our next task will be to estimate interactions between genetic background and genomic response to morphine in the four analysed inbred mouse strains. This approach would continue to expand research on the relationship between gene expression and specific opioid-related behaviours.

## Conclusion

Identification of entire gene networks and single genes that underlie or modify susceptibility to morphine would open new perspectives of pharmacological interventions. Here, we suggest involvement of several new genes in complex traits associated with opioid-related phenotype. The present results suggest strong contribution of genetic background to differences in gene expression. This observation is important for comparisons of gene expression studies carried on different inbred strains and interpretation of results from experiments performed on knock-out animals with mixed background. In summary, genomic factors with potential ability to underlie differences in the response to opioids are described in the presented paper. The identified genes provide promising candidates for future studies in animals and for translational genetic studies in the field of addictive and analgesic properties of opioids.

## Methods

### Tissue collection and RNA isolation

Adult male (8 to 10 weeks of age) 129P3/J (000690), DBA/2J (000671), C57BL/6J (000664) and SWR/J (000689) mice (The Jackson Laboratory, Bar Harbor, Maine, USA) were housed 6 per a cage, under 12 h dark/light cycle, with *ad libitum *access to food and water. Animals were familiarized with handling procedure for 5 days and sacrificed after the last session. All animals were drug-naïve at the time of sacrifice. After decapitation, brains were removed from the skulls and dissected rapidly. We collected samples containing rostral part of caudate/putamen plus the nucleus accumbens (referred to as the striatum). The samples were placed in individual tubes with tissue storage reagent RNA *later *(Qiagen Inc., Valencia, CA, USA), frozen on dry ice and stored at -70°C until RNA isolation. Samples were thawed at room temperature and homogenized in 1 ml of Trizol reagent (Invitrogen, Carlsbad, CA, USA). RNA isolation was performed according to the manufacturer's protocol. Quality of the total RNA was assessed by intensity of 28S and 18S bands after agarose electrophoresis with SybrGold staining (Molecular Probes, Inc., Eugene, Oregon, USA) and by spectrophotometric ratio A260/A280 (1.9–2.1). RNA concentration was measured using the fluorescent reagent RiboGreen (Molecular Probes, Inc., Eugene, Oregon, USA).

### Microarray hybridization

The same amount of total RNA from three animals was pooled, and further purified by using RNeasy Mini Kit (Qiagen Inc., Valencia, CA, USA). For each array independent pool of RNA from three animals was prepared. Sample pooling was performed to decrease variation within the group [59]. The quality of RNA was additionally determined by using RNA 6000 Nano LabChip Kit and Agilent Bioanalyzer 2100 (Agilent, Palo Alto, CA, USA), and there was little evidence of degradation products in any of the total RNA samples. Preparation of cRNA was performed according to the protocol provided by Affymetrix. Total RNA (5 μg) derived from each pool was converted to double-stranded cDNA using SuperScript System (Invitrogen, Carlsbad, CA) and an oligo(dT_24_) primer containing a T7 RNA polymerase promoter site (Genset Oligos, La Jolla, CA). Biotin-labelled cRNA was synthesized from cDNA using a BioArray High Yield RNA Transcript labelling Kit (ENZO, Diagnostics, Farmingdale, NY) and purified by using a GeneChip Cleanup Sample Module (Qiagen Inc., Valencia, CA, USA). Each cRNA sample was synthesized from independent biological sample. The yield of the in vitro transcription reaction was determined by product absorbance at 260 nm measured by NanoDrop ND-1000 (NanoDrop Technologies, Inc., Montchanin, DE), size of cRNA probes was evaluated by using RNA 6000 Nano LabChip Kit (Agilent, Palo Alto, CA, USA). Following labelling, samples were hybridized to the GeneChip Test3 array (Affymetrix, Santa Clara, CA) for quality control. Fragmented cRNA (15 μg) was used for hybridization to GeneChip Mouse Genome 430 2.0 arrays (Affymetrix, Santa Clara, CA). New generation of Mouse Genome Genechips, with improved quality of probes, was used to detect extended number of transcripts (45.101 probe-sets). Arrays were washed and stained with streptavidin-phycoerythrin (Merck, Darmstadt, Germany) in Fluidic Station 400 (Affymetrix, Santa Clara, CA), according to standard protocol of the manufacturer. The arrays were scanned by using GeneChip Scanner 3000 (Affymetrix, Santa Clara, CA). Results were obtained from arrays processed in two batches, in first batch 12 arrays were hybridized (3 independent replicates per each strain) and 8 in the second (2 independent replicates per strain). All the arrays were run using a single protocol. Five chips were prepared for each strain of mice.

### Microarray data analysis

Microarray data was initially processed using GeneChip Operating Software (GCOS, Affymetrix, Santa Clara, CA). Chip quality was assessed using R 2.1.0 with simpleaffy package [60,61]. Parameters such as scaling factor (0.64 ± 0.11), % of present call (51.7 ± 0.63) and 3'/5' degradation ratio (AFFX-GapdhMur/M32599 = 0.26 ± 0.22 and AFFX-b-ActinMur/M12481 = 0.8 ± 0.23) were compared according to Affymetrix guidelines. Raw data was normalized by multiple available methods using R 2.1.0 with Bioconductor with affy package, dChip 1.3 and PerfectMatch 2.3.0.0 software [61-64]. For selected genes signal from each probe was compared between all the arrays with dChip software to identify indications of SNPs in the region of probe-set hybridization. Full dataset with gene expression values generated by PDNN algorithm are included in additional material (see Additional file 1).

### Gene ranking and filtering

Significance levels (p-values) of differences between the four inbred strains were calculated for each probe-set using analysis of variance [65] for three pre-processing methods. In our opinion, traditional Bonferroni correction for multiple tests is too stringent for that kind of dataset, and would lead to passing over of important results. Therefore, false discovery rates (q-values) were calculated for ANOVA significance levels using q-value package for R [30]. For our purposes, we constructed a rank based on q-value levels of three selected methods (RMA, PDNN, MBEI). A probe-set scored 1, 2 or 3 points if it achieved cut-off at q < 0.01, q < 0.001 or q < 0.0001, respectively, for each of three methods. Every probe-set gets a rank from 0 to 9, where 9 means that probe-set was highly significant in all three normalization method, 3 means that probe-set was medium significant in all three methods or highly significant in only one method, 0 means that probe-set reached significance level in none of the three chosen methods (Figure 2). The proposed scheme, which takes into consideration results of three methods, requires a probe-set to be highly significant in one of the methods or less significant but in each of the methods to be selected for further analysis. The following criteria were applied for probe-set detection: present call at least in 25% of PM/MM pairs and signal intensity >6.64 (log_2 _data) in at least 25% of arrays, both measured by dChip software. Additionally, strain-specific differences in the basal gene expression were selected by computing between-strain contrast matrix for each probe-set using unpaired t-test with expression values normalized by PDNN. Gene expression level was considered to be specific for a particular strain if it was significantly different (p < 0.01) in all three contrasts given to the strain. To reliably reveal difference in low abundant transcripts, analyses were performed on five biological replicates of the microarray. Hierarchical clustering and measurement of relative expression levels were performed using dChip software and MBEI algorithm [63].

### Bioinformatic analysis of gene expression patterns

Gene sequences and exon structures were downloaded from GenBank and Ensembl databases, and analyzed for possible polymorphisms in the region covered by the probe-sets. Direct probe mapping against publicly available mRNAs/cDNA sequences was done by using mouse BLAT tool for UCSC genome browser and ADAPT database [66,67]. The Database for Annotation, Visualization and Integrated Discovery (DAVID 2.1), a functional annotation analysis tool, was used to identify over-represented ontological groups among gene expression profiles and to group genes into functional classifications [68]. Analyses were performed at level five and with default parameters which provide low coverage and relatively high specificity. Over-represented GO terms were defined as having p ≤ 0.01 under Fisher exact test. WebQTL database was used to link gene expression to genetic markers and to detect impact of regulatory variation [28].

### Analysis of correlation between gene expression and phenotypes

Relations between the inter-strain differences in transcriptional profile and two morphine-related traits were studied using correlation analysis. Results of voluntary morphine consumption in two-bottle choice paradigm (C57BL/6J = 134 mg/kg/day, 129P3/J = 24 mg/kg/day, DBA/2J = 15 mg/kg/day and SWR/J = 6 mg/kg/day) published by J. Belknap, 1993 [6] and analgesic effects of 3.6 mg/kg morphine in the hot-plate assay (129P3/J = 100% MPE, DBA/2J = 82% MPE, SWR/J = 56% MPE and C57BL/J6 = 7% MPE) estimated in our laboratory by W. Solecki (Solecki et al., in preparation) were used in the analysis. Correlations between the expression level from 1528 probe-sets (PDNN) and the phenotypic data was computed using the Pearson's correlation. Probe-sets with r > |0.95| and p < 0.05 were taken into further consideration. Separate analysis of interactions with the both traits was carried out using publicly available BXD IR strains database (BXD Published Phenotypes Database). All correlations were computed using HBP/Rosen Striatum M430 v2.0 (Apr05) PDNN Clean dataset. List of probe-sets with r > |0.5| and significant correlation with trait ID 10029 (morphine analgesia in response to 16 mg/kg morphine, hot-plate assay) and 10469 (morphine consumption-two bottle choice) were downloaded using WebQTL scriptable interface [69,70]. Analyses were determined using the Pearson's correlation with default parameters, significant correlation was found at p < 0.05. Lists of probe-sets with significant results obtained separately using both approaches were generated.

The list of genes regulated by opioid administration in the LC and VTA of C57BL/6J mice was built based on supplementary material published by McClung et al. [34]. Updated annotations for 94 probe-sets from the MG-U74Av2 microarray were obtained from Affymetrix website .

### Real-time PCR

Reverse transcription was performed with Omniscript RT enzyme (Qiagen Inc., Valencia, CA, USA) at 37°C for 60 min. Reaction was carried out in the presence of RNAse inhibitor (rRNAsin, Promega, USA), and oligo(dT_16_) primer (Qiagen Inc., Valencia, CA, USA) was used to selectively amplify mRNA. Quantitative real-time RT-PCR reactions were performed using Assay-On-Demand Taqman probes (details in Table 3, with the exception for *Grm7 *gene for which primers and probe were designed with Primer3 software) according to the manufacturer's protocol (Applied Biosystems, Foster, CA, USA), and run on the iCycler device (BioRad, Hercules, CA, USA) with the 3.0a software version. For each reaction ~50 ng of cDNA synthesized on total RNA template from individual animal were used. Entire qPCR experiment was performed twice, on samples used for array hybridization qPCR I (n = 6–9) and on independent RNA samples qPCR II (n = 6). To minimise the contribution of contaminating genomic DNA primers were designed to span exon junctions. Additionally, control reactions without RT enzyme for each assay were performed. Amplification efficiency for each assay was determined by running a standard dilution curve. Expression of hypoxanthine guanine phosphoribosyl transferase 1 (*Hprt1*) transcript with stable level between four strains was quantified to control for variation in cDNA amounts. The cycle threshold values were calculated automatically by iCycler IQ 3.0a software with default parameters. Abundance of RNA was calculated as 2^-(threshold cycle)^. Data was analyzed by one-way ANOVA followed by Tukey post hoc test.

## Authors' contributions

MK interpreted the data and participatedinthedesignofthestudy. DKC and MK carriedouttotalRNAisolation, sample preparation and qPCR analysis. MP and MK carried out bioinformatic analyses and wrote the manuscript. RP coordinated the study, carried out tissue preparation, aswellasreviewedthemanuscript. All authors read and approved the final manuscript.

## Supplementary Material

Additional File 2 List of probe-sets of genes differentially expressed among the four inbred strains of mice. Significance level for each probe-set is presented as rank and q-value. The next four sheets contain strain specific differences in the basal gene expression selected by computing between strains contrast matrix for significantly different probe-sets (selected by ANOVA) using unpaired t-test. Gene expression level was considered to be specific for a particular strain if it was significantly different (p < 0.01) in all three contrasts given for the strain. Click here for file

Additional File 1 Full dataset of gene expression values obtained by using PDNN algorithm. Table contains logged results from 45.101 probe-sets for each biological replicate, including signal from Affymetrix spike-in controls. Click here for file

Additional File 3 Detailed description of Gene Ontology analysis presented in Table 1 and Table 2. List of probe-sets and gene names classified to each GO category. Analyses were performed with DAVID 2.1 software. Click here for file

## Figures and Tables

**Table 1:** Functional classification of genes selected from microarray analysis.

**GO Category**	**GO Term (Term ID)**	**Count #genes**	**Significance p-value**
Biological Process	Protein Biosynthesis (GO:0006412)	42	0.002
Biological Process	Vesicle-Mediated Transport (GO:0016192)	25	0.002
Biological Process	Macromolecule Biosynthesis (GO:0009059)	45	0.003
Cell Component	Mitochondrion (GO:0005739)	64	0.004
Biological Process	Carboxylic Acid Metabolism (GO:0019752)	28	0.004
Biological Process	Cellular Protein Metabolism (GO:0044267)	155	0.004
Cell Component	Ribosome (GO:0005840)	22	0.006
Biological Process	Cytoskeleton Organization and Biogenesis (GO:0007010)	31	0.007
Biological Process	Cellular Lipid Metabolism (GO:0044255)	28	0.007
Cell Component	Small Ribosomal Subunit (GO:0015935)	7	0.009
Biological Process	Small Gtpase Mediated Signal Transduction (GO:0007264)	21	0.010
Biological Process	Synaptic Transmission (GO:0007268)	15	0.010

Over-represented functional classes of genes among transcripts with different expression profiles between the mouse strains were found using DAVID 2.1 tool. Analysis were performed at level five and with default parameters which provide low coverage and relatively high specificity for detection of enriched terms. Over-represented GO groups were found at p ≤ 0.01 level Fisher exact test.

**Table 2:** Functional classification of strain-specific gene expression.

**C57BL/6J**			
Biological Process	Negative Regulation of Cell Organization and Biogenesis (GO:0051129)	4	<0.001
Biological Process	Regulation of Microtubule Polymerization or Depolymerization (GO:0031110)	3	0.006
Biological Process	Cytoskeleton Organization and Biogenesis (GO:0007010)	11	0.008
Biological Process	Cellular Lipid Metabolism (GO:0044255)	10	0.010

**DBA/2J**			

Biological Process	Cellular Lipid Metabolism (GO:0044255)	11	<0.001
Biological Process	Carboxylic Acid Metabolism (GO:0019752)	9	0.004
Cell Component	Microbody (GO:0042579)	5	0.005
Cell Component	Peroxisome (GO:0005777)	5	0.005

**129P3/J^a^** **SWR/J**			

Biological Process	Regulation of Apoptosis (GO:0042981)	9	0.002
Biological Process	Regulation of Programmed Cell Death (GO:0043067)	9	0.002
Biological Process	Negative Regulation of Programmed Cell Death (GO:0043069)	9	0.009

^a ^no significantly over-represented groups were found for 129P3/J strain

**Table 3:** Gene expression results obtained by microarrays and qPCR.

**Gene**	**Affy ID PCR assay ID (exon boundary)**	**Strain**	**Microarray (MBEI)^a ^(n = 5)**		**qPCR I^b ^(n = 6–9)**	**qPCR II^c ^(n = 6)**
					
			**1^d^**	**2**		
ATPase, Na+/K+ transporting, alpha 2 polypeptide (*Atp1a2*)	(1)1427465_at (2)1455136_at Mm00617899_m1 (20/21)	**C57**	1.24 ± 0.08	**0.52 ± 0.08**	1.15 ± 0.07	1.19 ± 0.12
		**DBA**	0.98 ± 0.07	1.39 ± 0.1	0.9 ± .0.06	0.96 ± 0.17
		**129**	0.9 ± 0.06	1.08 ± 0.06	0.89 ± 0.1	0.83 ± 0.15
		**SWR**	0.88 ± 0.07	1.02 ± 0.05	1.06 ± 0.09	0.98 ± 0.07
Catechol-O-methyltransferase (*Comt*)	1418701_at Mm00514377_m1 (4/5)	**C57**	**0.48 ± 0.08**		1.48 ± 0.08	**1.59 ± 0.06**
		**DBA**	1.17 ± 0.04		0.93 ± 0.05	0.9 ± 0.08
		**129**	1.19 ± 0.07		0.89 ± 0.05	0.78 ± 0.07
		**SWR**	1.16 ± 0.11		0.71 ± 0.09	0.76 ± 0.08
Gamma-aminobutyric acid receptor (GABA-A), subunit alpha 1 (*Gabra1*)	1436889_at Mm00439040_m1 (2/3)	**C57**	1.09 ± 0.03		**1.62 ± 0.1**	1.46 ± 0.1
		**DBA**	0.76 ± 0.02		0.74 ± 0.24	0.82 ± 0.04
		**129**	1.09 ± 0.05		0.81 ± 0.31	0.96 ± 0.08
		**SWR**	1.06 ± 0.04		0.83 ± 0.33	0.78 ± 0.03
Gamma-aminobutyric acid receptor (GABA-A), subunit alpha 2 (*Gabra2*)	(1)1443865_at (2)1455444_at Mm00433435_m1 (5/6)	**C57**	**0.55 ± 0.06**	**0.57 ± 0.03**	**0.43 ± 0.07**	**0.26 ± 0.08**
		**DBA**	1.08 ± 0.11	1.09 ± 0.07	1.26 ± 0.09	1.13 ± 0.08
		**129**	1.2 ± 0.07	1.21 ± 0.04	1.25 ± 0.08	1.31 ± 0.07
		**SWR**	1.17 ± 0.07	1.13 ± 0.05	1.06 ± 0.1	1.05 ± 0.05
Glutamate receptor, metabotropic 7 (*Grm7*)	1459532_at Primers and probe sequence are available on request (2)	**C57**	**0.46 ± 0.14**		1.09 ± 0.11	1.03 ± 0.13
		**DBA**	**2.86 ± 0.16**		0.73 ± 0.13	0.66 ± 0.32
		**129**	**0.32 ± 0.14**		1.46 ± 0.11	1.21 ± .013
		**SWR**	**0.36 ± 0.14**		0.72 ± 0.18	1.00 ± 0.12
Potassium inwardly-rectifying channel, subfamily J, member 9 (*Kcnj9*)	1450712_at Mm00434623_m1 (3/4)	**C57**	**0.42 ± 0.13**		**0.48 ± .0.08**	**0.51 ± 0.16**
		**DBA**	1.18 ± 0.12		1.23 ± 0.05	1.25 ± 0.11
		**129**	0.94 ± 0.06		1.16 ± 0.07	1.15 ± .0.08
		**SWR**	1.46 ± 0.11		1.13 ± 0.05	1.15 ± 0.08
Synaptotagmin IV (*Syt4*)	1415844_at Mm00436870_m1 (1/2)	**C57**	1.33 ± 0.01		nt^e^	**1.51 ± 0.18**
		**DBA**	1.21 ± 0.07		nt	1.06 ± 0.03
		**129**	0.71 ± 0.08		nt	0.86 ± 0.23
		**SWR**	0.75 ± 0.06		nt	**0.61 ± 0.13**
Glial fibrillary acidic protein (*Gfap*)	1440142_s_at Mm01253033_m1 (6/7)	**C57**	1.29 ± 0.09		1.29 ± 0.12	1.12 ± 0.08
		**DBA**	0.78 ± 0.12		0.88 ± 0.11	0.88 ± 0.06
		**129**	**0.5 ± 0.07**		0.7 ± 0.13	0.78 ± 0.11
		**SWR**	1.43 ± 0.05		1.12 ± 0.07	1.14 ± 0.07
Microtubule-associated protein 2 (*Mtap2*)	1440699_at Mm00485230_m1 (3/4)	**C57**	**0.62 ± 0.15**		nt	0.94 ± 0.14
		**DBA**	1.12 ± 0.05		nt	1.03 ± 0.21
		**129**	1.22 ± 0.03		nt	1.19 ± 0.18
		**SWR**	1.13 ± 0.05		nt	0.84 ± 0.26
Hypoxanthine guanine phosphoribosyl transferase 1 (*Hprt1*)	1448736_a_at Mm00446968_m1 (6/7)	**C57**	1.03 ± 0.04		1.09 ± 0.09	1.1 ± 0.1
		**DBA**	1.01 ± 0.03		1.02 ± 0.04	0.93 ± 0.08
		**129**	1 ± 0.02		1 ± 0.08	1.02 ± 0.04
		**SWR**	0.97 ± 0.04		0.9 ± 0.09	0.88 ± 0.11

Results are presented as fold change of average expression level across all the four mouse strains ± standard error of the mean. Results with fold change above 1.5 are bolded. Taqman used in qPCR experiments are described by assay ID and exon boundary.

^a ^relative expression levels from probe-sets significantly different between the strains were obtained by using MBEI algorithm.

^b ^qPCR I was carried out on individual samples used also for microarray hybridization

^c ^qPCR II was performed on independent samples

^d ^two genes (*Atp1a2 *and *Gabra2*) are represented by more than one probe-set.

^e ^nt – gene was not tested
